# Supplementary material for: Overexpression of a Poplar RING-H2 Zinc Finger, Ptxerico, Confers Enhanced Drought Tolerance via Reduced Water Loss and Ion Leakage in Populus
Source: Int J Mol Sci. 2020 Dec 11;21(24):9454. doi: 10.3390/ijms21249454 (PMC7764267; doi:10.3390/ijms21249454)
Supplement: Supplementary file 1 [file ijms-21-09454-s001.pdf]

Supplemental Data

Supplemental Table S1. Primer sequences used in this study.

Supplemental Figure S1. Leaf morphology and chlorophyll contents of 35S::PtrXERICO Arabidopsis plants. (a) Forty-day-old rosette leaves of WT, 35S::PtrXERICO and 35S::AtXERICO plants. Scale bar, 1cm. (b) Leaf size were measured from (a). (c) Chlorophyll contents (a + b) were measured. Error bars indicate standard error (n = 10).

Supplemental Figure S2. Root growth of 35S::PtrXERICO Arabidopsis plants by salt stress. Root growths were measured from the vertically grown plants with or without 100 mM NaCl in the MS-agar medium. Error bars indicate standard error of three independent experiments.

Supplemental Table S1. Primer sequences used in this study.

Gene ID	Gene Name	Primers	Sequence (5' ~ 3')	Size (bp)
Constructs for producing transgenic plants				
Potri.014G170400	*PtXERICO*	Forward	aaaaagcaggctATGGGTCTATCAAGTCTGCCA	488
		Reverse	agaaagctgggtTCACCAAAAGCAAGATGCA	
		35S promoter	TATCCTTCGCAAGACCCTTCCTC	688
		attB1	CAAGTTTGTACAAAAAAGCAGGCT	540
		attB2	ACCACTTTGTACAAGAAAGCTGGGT	
For semi-quantitative RT-PCR				
AT1G49240	*AtActin8*	Forward	ATGAAGATTAAGGTCGTGCA	418
		Reverse	TCCGAGTTTGAAGAGGCTAC	
Potri.014G170400	*PtXERICO*	Forward	ATGGGTCTATCAAGTCTGCCA	462
		Reverse	TCACCAAAAGCAAGATGCA	
AT2G04240	*AtXERICO*	Forward	ATGGGTCTATCAAGTCTTCCTGGT	489
		Reverse	TCACCAAACATTAGAAGAAAGCTG	
	AtXERICO_UTR	Reverse	TGTGTTCAAACAAGAGCTCCA	662
AT4G08040	*AtACS11*	Forward	TTCTTATCCCTGCACCTTATTATCC	977
		Reverse	TCAACGTTCTGATTCACAAGTAACA	
AT3G14440	*AtNCED3*	Forward	AACTTCGTCGTCGTACCTGA	737
		Reverse	ACCTGCTTCGCCAAATCAT	
AT2G29090	*AtCYP707A*	Forward	ATTGCTGACAACATCATCGG	596
		Reverse	ATCGGGGTTACTCTTATTGG	
AT5G52310	*AtRD29A*	Forward	GAACACTCCGGTCTCTCTGC	800
		Reverse	ATCCAGGTCTTCCCTTCGCC	
For quantitative RT-PCR				
Potri.014G170400	*PtXERICO*	Forward	CATCAATCCGATTCAACACG	199
		Reverse	TCTTCAGGCAGCAAAGGAGT	
Potri.011G112400	*PtNCED3*	Forward	AGTCGCCCAATGTTGAAATC	200
		Reverse	GGAAGCATCAGTGGCATTCT	
Potri.001G242600	*PtCYP707A2*	Forward	GAGTGATTTTTGCCGCTCAT	200
		Reverse	TCTCTTGGATCACCCGACTT	
Potri.002G113900	*PtACS11*	Forward	GGACCGACACCTGGAAAATA	199
		Reverse	AGAAAGCATGCCCGAGAGTA	
Potri.019G010400	*PtActin2*	Forward	GCCATCTCTCATCGGAATGGAA	171
		Reverse	AGGGCAGTGATTTCCTTGCTCA	

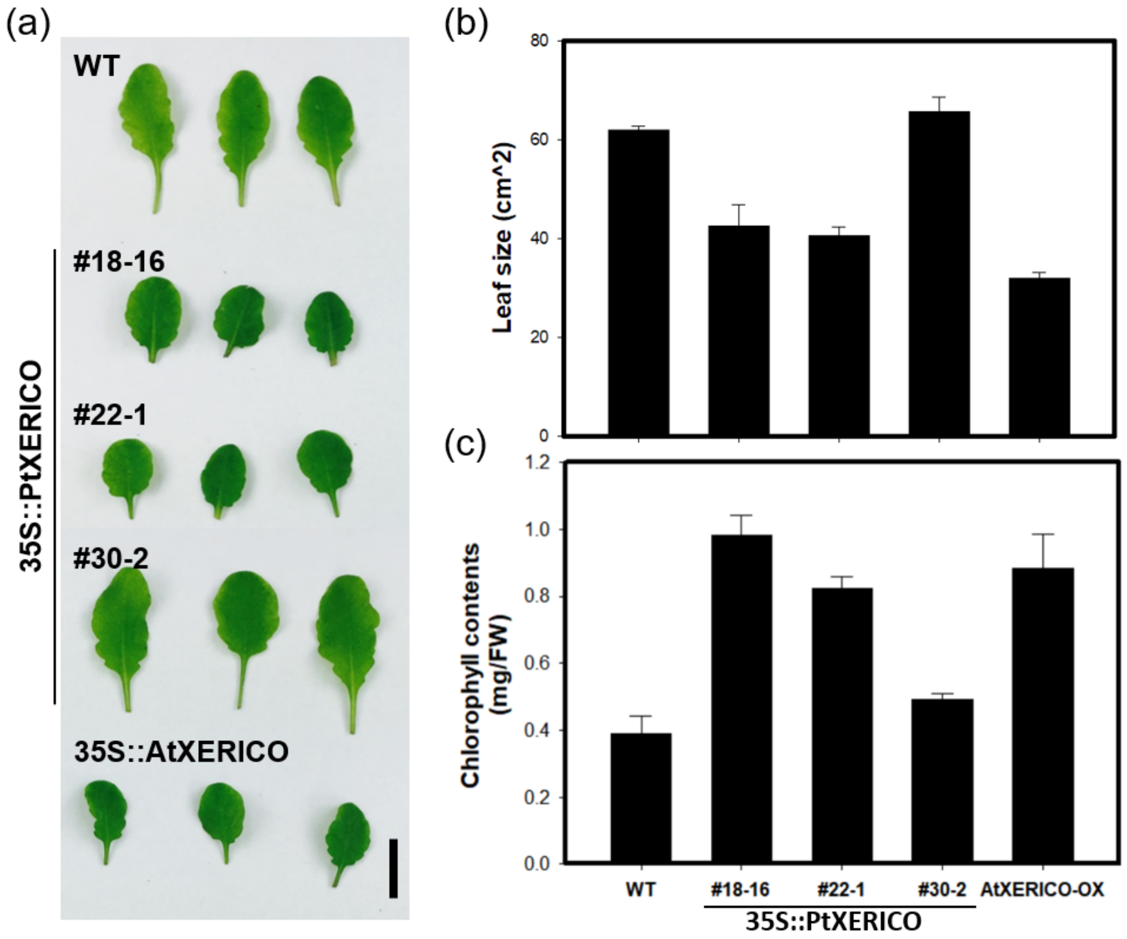

Supplemental Figure S1. Leaf morphology and chlorophyll contents of 35S::PtrXERICO Arabidopsis plants. (a) Forty-day-old rosette leaves of WT, 35S::PtrXERICO and 35S::AtXERICO plants. Scale bar, 1cm. (b) Leaf size were measured from (a). (c) Chlorophyll contents (a + b) were measured. Error bars indicate standard error (n = 10).

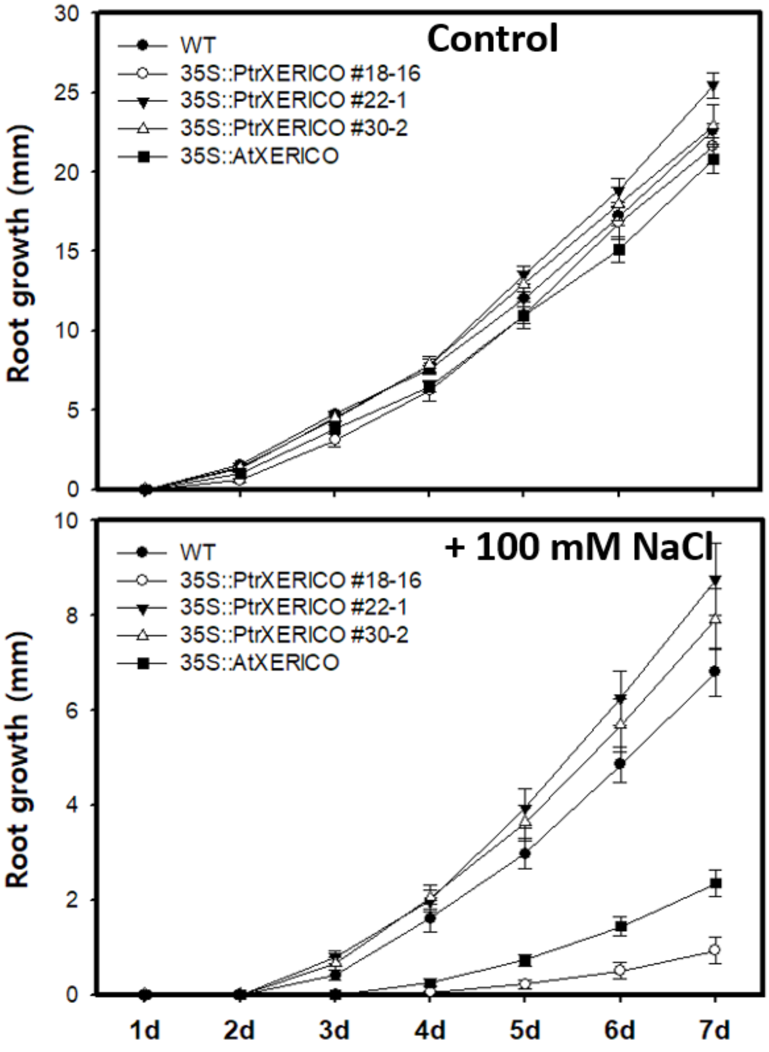

Supplemental Figure S2. Root growth of 35S::PtrXERICO Arabidopsis plants by salt stress. Root growths were measured from the vertically grown plants with or without 100 mM NaCl in the MS-agar medium. Error bars indicate standard error of three independent experiments.
